# The Treatment of Pneumonia*Paper read before Atlanta Society of Medicine, Tuesday, February 4th.

**Published:** 1896-03

**Authors:** James B. Baird

**Affiliations:** Atlanta, Ga.


### THE TREATMENT OF PNEUMONIA.*

By JAMES B. BAIRD, M.D.,

Atlanta, Ga.

The preponderance of evidence seems to sustain the view that acute lobar pneumonia is a general infectious disease, dependent for its production upon a specific causative agent, with a local expression represented by an exudative inflammation of the pulmonary parenchyma, rather than a local inflammatory lesion with associated symptomatic phenomena.

The natural history of this affection establishes the fact that it is a self-limited disease and, in uncomplicated cases, under favorable conditions, tends toward recovery.

If these propositions are accepted, the study of the therapeutics of pneumonia is simplified, and the indications for treatment are more easily read aright.

It is doubtful if we possess an abortive remedy. Half a century ago when active antiphlogistic doctrines were held in high esteem, general blood-letting was thought to possess no abortive influence, and this measure was assiduously employed.

It is now generally, perhaps universally, conceded that venesection is not abortive or curative and that even as a mere palliative the range of its usefulness is extremely limited.

During the first stage of an attack, in a robust, so-called plethoric subject, suffering from distressing dyspnea, with a full, bounding pulse and excessive pyrexia, the moderate abstraction of blood from the arm is admissible, and it affords immediate relief from the painful symptoms, both by a reduction of the total volume of blood and by removing the disturbing pressure from the overcharged lungs and from an overburdened heart.

The objections to its use relate to its spoliative effect upon the vital fluid in a disease which, in tedious, intense, or unfavorable cases, tends to destroy life by asthenia. The remarkable and perhaps too radical change which has taken place in respect to this

---

*Paper read before Atlanta Society of Medicine, Tuesday, February 4th.

measure of treatment within the last fifty or sixty years is not explained by any change in the type of disease, but rather by increased knowledge and by the possession of new therapeutic resources, which, while they do not act so promptly, accomplish with a little delay, practically the same results without any ulterior damaging consequences to the vital forces.

It is perhaps proper to state that in an active practice covering a period of twenty-five years, with a fair opportunity for observation and experience, I have never met with a case in which I considered blood-letting necessary or desirable.

It is always difficult to correctly estimate the value of preventive medication, but trustworthy testimony ascribes to quinine the power to arrest a certain proportion of cases of acute lobar pneumonia if administered in full doses early in the first stage. Thus used the remedy possesses the negative merit of being innocuous, even should it fail to meet sanguine expectations.

In the management of pneumonia it is profitable to bear in mind the trite maxim, that the patient and not the disease should be treated. So, with this restraining influence at hand, the resources of our art may best be adapted to the variable and the varying conditions of the patient.

The general indications call for the internal and the external use of remedies, for the relief of pain, allaying cough, reducing pyrexia, procuring rest, sustaining the vital forces, and establishing a tolerance of the disease.

These indications, it will be observed, are based upon the prominent symptoms of the disease, and it is therefore, to the symptoms that our treatment should be addressed, for it must be admitted that, in the present state of our knowledge, we have no specific remedy capable of aborting, arresting, or abridging the attack. The treatment is consequently, for the most part, expectant, but let it be distinctly understood that this term is not synonymous with do-nothing; on the contrary, it is not incompatible with the employment of the most energetic and direct measures.

To relieve pain and subdue cough, opium in some form is the most potent remedy. It should be given in such doses and at such intervals as may be requisite to accomplish these objects. In this, as in other inflammatory affections, it is not only palliative, but is positively curative. Without entering into a full review of its mode of action, suffice it to say, that it conserves the powers of life and promotes a tolerance of painful and perturbing disease.

Excessive heat may be combated by the administration of antipyretics and by the external application of cold. These remedies are usually indicated if the temperature reaches 103° Fahrenheit. Of all the antipyretic drugs, I prefer phenacetin. In proper cases I consider its use not only admissible but advisable. I take this position with a full recognition of the fact that it is at variance with opinions and practices of many able men. I believe it can be easily sustained, and some of the arguments urged against the practice appear to me fanciful and purely theoretical. According to my judgment, it is only in cases manifesting marked cardiac depression that it should be withheld. It may then be substituted by the bath, wet pack, or by sponging. If it is not alone effective, it may be supplemented by the sponge bath.

I do not attempt to obtain the antipyretic effect of quinine. If the disease progresses in spite of a few full doses of the drug at the outset, it should be laid aside except in moderate doses. I believe it is often an advantage to exhibit quinine and phenacetin combined, in doses of two or three grains of each, three or four times daily for several days during the acute period of the attack, and for this purpose I prefer the bisulphate to the ordinary sulphate salt.

My experience with the immersion bath, the cold pack, the coils, or the ice bags does not enable me to speak with confidence regarding their utility or their superiority over other means. The popular prejudice against the application of cold in a disease generally supposed to depend upon cold as a causative influence, is worthy of consideration. Sponging, or even partial sponging, of the readily accessible surface of the body with tepid water or alcohol is to be commended and is often surprisingly efficient. I discard the general application of cold water because it is usually disagreeable to the patient and causes more or less shock.

Sound sleep and mental and physical quietude are desirable; and especially during the acute stage, one of the bromides, aided, if necessary by small additions of chloral at night, usually yields happy results.

Judicious support is indispensable. This is accomplished by appropriate alimentation, by stimulants and by cardiac tonics. The food should be for the most part liquid, and of sufficient quantity. Milk, broth, animal essences, and eggs, with some farinaceous articles, should be allowed.

Alcoholic stimulants are indicated in the vast majority of cases. It is a grave mistake to defer their use too long. Recognizing the general tendency of the disease towards an asthenic state, the necessity for their employment may be profitably anticipated, and thus being forewarned our patient may be forearmed. The pulse affords a safe guide to the dose. Both the quality and the frequency of the pulse is to be considered. I usually begin the administration in an adult with half an ounce three or four times daily in milk or water, and, noting the effect, gradually or promptly increase the amount prescribed as required, giving, in extreme cases, as much as an ounce every hour or two. In my judgment this apparently heroic practice may sometimes turn the tide and avert disaster. The carbonate or the aromatic spirit of ammonia may prove a valuable adjunct, but in full doses, long continued, they are apt to derange the stomach. For the support of the heart, I consider strychnine and digitalis the most reliable remedies at our command. One or both should always be given when signs of cardiac weakness become manifest and persist despite the alcoholic stimulation.

In cases presenting a full bounding pulse and distressing dyspnea, veratrum viride or aconite is very grateful and is unquestionably serviceable. I consider the cautious use of these drugs entirely safe and, in appropriate cases, to be recommended. They are not used of late years as frequently as formerly, but they have their place in the therapeutics of acute pneumonia, and they should not be discredited.

Cathartics are usually indicated at the commencement and from time to time during the career of the disease. Saline laxatives in many instances act well, but I know that calomel is a much more thorough eliminant than this class of remedies and is, in consequence, often of greater service.

I do not recommend or approve of blisters in the earlier stages of the disease. They increase the discomfort of the patient and seriously interfere with satisfactory physical exploration, besides, in my judgment, being powerless for good. Mild counter-irritants are unobjectionable and generally suffice to alleviate if not to allay the pleuritic pain which is sometimes present in a conspicuous degree. Blisters, however, are not to be entirely rejected, for in the last stage, if the process of resolution or absorption should be arrested or unduly prolonged, cantharidal collodion or cerate over the affected part exerts a most favorable influence by stimulating the activity of nature's forces.

Under these circumstances sorbefacients are of doubtful utility. Mecurialization, I presume, now has no advocates.

The oil-silk jacket indorsed and employed by many practitioners is probably moderately beneficial by promoting gentle diaphoresis, but, like blisters, it prohibits physical examination of the chest.

During convalescence general tonic and hygienic measures are appropriate.

The development of secondary or intercurrent affections, or of complications during the course of pneumonia, should be met by treatment appropriate to the complication irrespective of the coexistent pneumonia, except in so far as the presence of the pneumonia may make it expedient in certain cases to modify the usual treatment of the complication.

---

### LUMBAGO.

A prescription of Dr. S. Solis-Cohen's is :—

| | |
|---|---|
| Sodium salicylate | ½ oz. |
| Potassium iodide | 2 dr. |
| Compound syrup of sarsaparilla | 1½ fl. oz. |
| Water, sufficient to make | 3 fl. oz. |

M. Sig.— A teaspoonful in water thrice daily, after meals.—*Pract. Med.*

